# Radiation-Induced Oral Mucositis

**DOI:** 10.3389/fonc.2017.00089

**Published:** 2017-05-22

**Authors:** Osama Muhammad Maria, Nicoletta Eliopoulos, Thierry Muanza

**Affiliations:** ^1^Faculty of Medicine, Experimental Medicine Department, McGill University, Montreal, QC, Canada; ^2^Radiation Oncology Department, Jewish General Hospital, McGill University, Montreal, QC, Canada; ^3^Lady Davis Institute for Medical Research, Jewish General Hospital, McGill University, Montreal, QC, Canada; ^4^Faculty of Medicine, Surgery Department, McGill University, Montreal, QC, Canada; ^5^Oncology Department, McGill University, Montreal, QC, Canada

**Keywords:** chemotherapy, oral mucositis, radiation, radiotherapy, normal tissue injury, pathobiology, mesenchymal stromal/stem cells

## Abstract

Radiation-induced oral mucositis (RIOM) is a major dose-limiting toxicity in head and neck cancer patients. It is a normal tissue injury caused by radiation/radiotherapy (RT), which has marked adverse effects on patient quality of life and cancer therapy continuity. It is a challenge for radiation oncologists since it leads to cancer therapy interruption, poor local tumor control, and changes in dose fractionation. RIOM occurs in 100% of altered fractionation radiotherapy head and neck cancer patients. In the United Sates, its economic cost was estimated to reach 17,000.00 USD per patient with head and neck cancers. This review will discuss RIOM definition, epidemiology, impact and side effects, pathogenesis, scoring scales, diagnosis, differential diagnosis, prevention, and treatment.

## Definition

**Figure 1:**
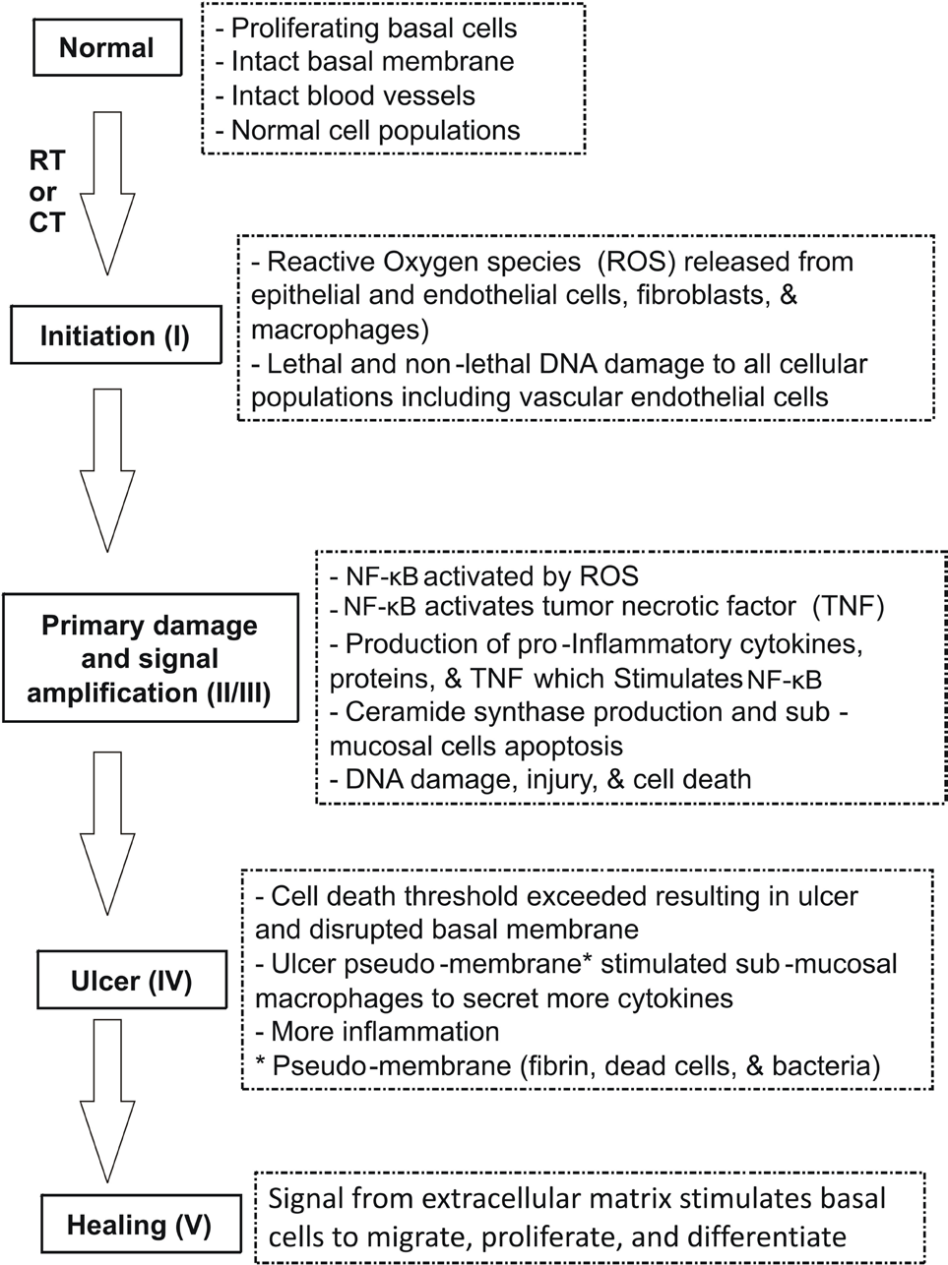
**Pathobiology of oral mucositis (OM) (10)**. Sonis has suggested five stages (phases) of OM injury induced by radiotherapy (RT) and/or chemotherapy (CT): initiation, signaling, amplification, ulceration, and healing. The pathogenesis of each phase is illustrated.

Radiation-induced oral mucositis (RIOM) (Figures 1, 4 and 5C) is one of the major ionizing radiation toxicities and normal tissue injuries that result from radiotherapy (1). RIOM was first termed in 1980 as a side effect of radiotherapy (RT) in cancer patients (2). RIOM is a normal tissue injury lasting between 7 and 98 days, which starts as an acute inflammation of oral mucosa, tongue, and pharynx after RT exposure (1, 3). This coincides with recruitment of various inflammatory cells and release of inflammatory cytokines, chemotactic mediators, and growth factors. RIOM can progress to an acute life-threatening stage as a result of severe physical obstruction of food and water intake with subsequent weight loss and septic complication due to lost protective epithelial and basement membrane barriers. This leads to limitations of local tumor control due to cancer treatment interruption and alterations in radiation dose fractionation (4–7). Studies suggested the stages of progression of RIOM as initial hyperemia and erythema during the preulcer phase, during which there is a release of various pro-inflammatory cytokines from epithelial, vascular, and connective tissue cells at the site of tissue injury. This is followed by the epithelial phase with various degrees of desquamation and basement membrane damage with loss of the protective barrier, which ends with the physical appearance of the ulceration. The postulcerative phase varies depending on the extent of the tissue toxicity. A secondary infection with Gram-negative bacteria or yeast may occur, with microcoagulation of the vasculature that worsens the inflammation by the local ischemia with more necrotic tissue yield. The final stage will be the healing phase and fibrosis (2, 8, 9).

## RIOM Epidemiology (Incidence, Predictors, and Risk Factors)

Radiation-induced oral mucositis occurs in up to 80% of head and neck cancer irradiated patients and reaches up to 100% in patients with altered fractionation head and neck cancer. RIOM of grade 3 and 4 have been recorded in 56% of head and neck cancer patients treated with radiotherapy (1, 12).

Many risk factors have been identified for RIOM. These risk factors include concomitant chemotherapy (CT), bad oral hygiene, below average nutritional stratus, lack of antibiotic use at early stage mucositis, and smoking (13).

Table 1 shows the significant predictors for the prevalence of severe RIOM and the symptoms of RIOM in a longitudinal study of patients with oral cavity cancer among head and neck cancer outpatients of a radiation department at a major medical center in Taiwan (14). This study used the Generalized Estimating Equations to analyze the predictive factors of prevalence of severe RIOM and RIOM-related symptoms. They found that the significant predictors for the prevalence of severe RIOM included type of treatment [RT vs. concomitant chemoradiotherapy (CCRT)], cumulative radiation dose, smoking, and body mass index (BMI). Patients who received CCRT (Coef. 0.145, *p* < 0.05), who have a higher cumulative radiation dose (Coef. 0.000, *p* < 0.01), who are smokers (Coef. 0.090, *p* < 0.01), and who have lower BMI (Coef. 0.005, *p* < 0.05) were at high risk to develop severe RIOM. RIOM-related symptoms were also predicted by type of treatment (RT vs. CCRT) (Coef. 1.618, *p* < 0.05), cumulative radiation dose (Coef. 0.003, *p* < 0.05), and smoking (Coef. 1.759, *p* < 0.001). These significant predictors are implemented by radiation oncologists to minimize and/or prevent the RIOM. June Eilers and Rita Million have summarized the patient-linked factors leading to increased risk for RIOM (Table 2) (15). They found that very young age, female gender, poor oral health and hygiene, decreased saliva secretion, low BMI, poor renal function with elevated serum creatinine level, smoking, and history of RIOM are risk factors predicting the development of RIOM in head and neck cancer patients (15).

**Table 1:** **Data analysis for RIOM predictors using IBM SPSS version 21.0 (Armonk, NY, USA) (14)**.

Variable	Coef.	SE	z	*p* > z	95% conf. interval	
**Prevalence of severe RIOM**						
Treatment type (RT vs. CCRT)	0.145	0.06	5.65	0.017	0.03	0.26
Cumulative RT dose (cGy)	0.000	0.03	16.47	0.001	−0.00	0.01
Smoking (no vs. yes)	0.090	0.03	8.52	0.004	0.03	0.15
Body mass index	−0.005	0.01	4.56	0.033	−0.10	0.00
Time	0.417	0.09	23.56	0.001	0.25	0.59
Intercept	−0.277	0.17	2.72	0.099	−0.61	0.05
**Symptoms of RIOM**						
Treatment type (RT vs. CCRT)	1.618	0.49	10.76	0.001	0.65	2.59
Cumulative RT dose (cGy)	0.003	0.01	4.03	0.045	−0.01	−0.01
Smoking (no vs. yes)	1.759	0.41	18.50	0.000	0.96	2.56
Body mass index	−0.002	0.05	0.00	0.973	−0.09	0.09
Time	1.338	1.34	2.57	0.109	−0.48	4.77
Intercept	6.023	2.77	4.74	0.030	0.60	11.45

*Significant predictors for RIOM*.

*Significant predictors for the prevalence of severe RIOM (CCRT, cumulative radiation dose, smoking, and low BMI) and the symptoms of RIOM in a longitudinal study of patients with oral cavity cancer among head and neck patients (CCRT, cumulative radiation dose, and smoking)*.

*CCRT, concomitant chemoradiotherapy; RIOM, radiation-induced oral mucositis*.

**Table 2:** **Patient-linked factors leading to increased risk for oral mucositis (OM) (15)**.

Age	Increased risk in very young age (high cell turnover rate) and old age (slower healing rate)
Gender	Trends to increased risk in females
Oral health and hygiene	Maintaining good oral hygiene and oral health lowers radiation-induced oral mucositis (RIOM) risk
Salivary secretory function	Decreased saliva leads to increased RIOM risk
Genetic factors	Potential for high RIOM risk in certain individuals still to be identified
Body mass index	Delayed healing and increased breakdown in malnourished individuals
Renal function	Increased mucotoxicity linked with high serum creatinine level (poor renal function)
Smoking	Delays the healing
Previous cancer treatment	History of mucositis due to previous cancer treatment increases the risk

## RIOM Impact and Side Effects

Radiation-induced oral mucositis side effects and sequels include oral pain in 69% of patients, dysphagia in 56% of patients, opioid use in 53% of patients, weight loss of 3–7 kg, feeding tube insertion and hospitalization (ICU admission) in 15% of patients, and modification or interruption of treatment in 11–16% of patients (1, 12, 16).

In the United States, RIOM may add up to 1,700.00–6,000.00 USD per patient depending on the inflammatory grade of the injury (12). RIOM treatment adds an economic cost that was estimated to increase up to 17,000.00 USD per patient treated for head and neck cancers (16).

Radiation-induced oral mucositis injury challenges radiation oncologists from many aspects, such as radiation dose limitations, changes in dose fractionation protocol, and dramatic negative effects on patients’ quality of life (1). The major clinical consequences of RIOM include hospital admission or extended hospitalization for total parenteral nutrition, intravenous (IV) analgesia, and IV antibiotics. Sixty-two percent of patients require hospitalization, and 70% of patients with grade 3–4 oral mucositis (OM) require feeding tube insertion. Reduction or cessation of cancer treatment occurs in 35% of patients due to the developed dose-limiting toxicity (17).

## Pathogenesis and Suggested Mechanistic Pathways

The pathophysiology of RIOM is not fully understood. Recent studies proposed that the pathogenesis of RIOM is composed of four phases: an initial inflammatory/vascular phase, an epithelial phase, a (pseudomembranous) ulcerative/bacteriological phase, and a healing phase (2, 5).

At the inflammatory phase, the RT-induced tissue injury results in the release of inflammatory cytokines; e.g., interleukin (IL)-1β, prostaglandins (PGs), and tumor necrosis factor-α (TNF-α) from the resident cells such as epithelial, endovascular, and connective tissue. These mediators might increase the damage by increasing the vascular permeability, leading to more infiltration and recruitment of inflammatory cells. Stem cells travel to the site of the tissue injury with other innate immunity components, e.g., MPO-positive leukocytes, macrophages, and neutrophils (18). On the other hand, there are some anti-inflammatory cytokines, such as IL-10 and IL-11, that work to minimize the injury (18).

The epithelial phase is initiated within a week by the apoptotic and cytotoxic effects of RT on the proliferating basal cells. This is why the recovery period is dependent on the rate of epithelial turnover, which could be enhanced by growth factors like epidermal growth factor and keratinocyte growth factor (KGF) (19).

After a week, the epithelial breakdown ends with the beginning of the ulceration. This occurs, when epithelial loss leads to disrupted basement membrane, formation of ulcer pseudomembrane, and inflammatory exudate. The ulceration stage is very painful, since the protective barrier that covers the nerve endings at the lamina propria is lost (19). The resulting microcoagulation and neutropenic state facilitate the Gram-negative bacteria and yeast colonization with the production of secondary infection. Bacterial exotoxins aggravate the inflammatory reaction by inducing mononuclear burst with the release of more IL-1β, TNF-α, and nitric oxide (8, 9, 16, 20).

Signaling pathways suggested to be involved in RIOM pathobiology include nitrogen metabolism, Toll-like receptor signaling, nuclear factor-κB (NF-κB) signaling, B-cell receptor signaling, P13K/AKT signaling, cell cycle: G2/M DNA damage checkpoint receptor, p38 mitogen-activated protein kinase (MAPK) signaling, Wnt/B-catenin signaling, glutamate receptor signaling, integrin signaling, vascular endothelial growth factor signaling, IL-6 signaling, death receptor signaling, and SAPK/JNK signaling (Table 3) (10, 19).

**Table 3:** **Signaling pathways involved in the development of mucositis (10)**.

B-cells receptor signaling
Cell cycle: G2/M DNA damage checkpoint receptor
Death receptor signaling
Glutamate receptor signaling
Interleukin-6 signaling
Integrin signaling
Nuclear factor-κB signaling
Nitrogen metabolism
PI3K/AKT signaling
P38 mitogen-activated protein kinase signaling
SAPK/JNK signaling
Toll-like receptor signaling
Vascular endothelial growth factor signaling
Wnt/B-catenin signaling

In 2004, Sonis suggested five stages (phases) of OM injury induced by radiotherapy (RT) and/or CT: initiation, signaling, amplification, ulceration, and healing (Figure 1) (16).

**Figure 2:**
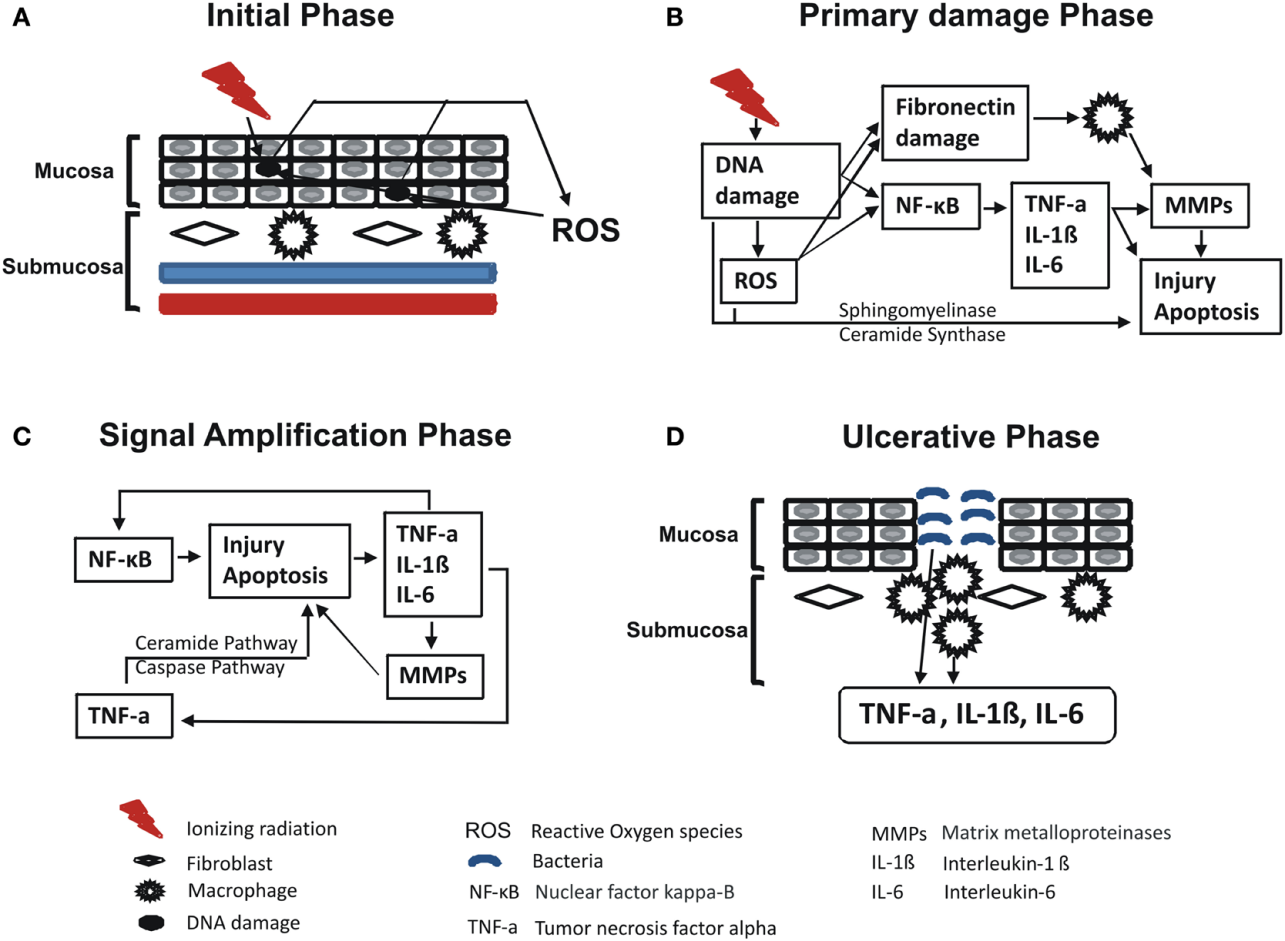
**Redding’s summary of RT and/or chemotherapy (CT)-induced oral mucositis pathobiology (11)**. Redding has summarized the pathobiology phases of radiation-induced oral mucositis induced by RT and/or CT. In brief, initiation phase with RT and/or CT results in direct and lethal DNA damage, which leads to release of reactive oxygen species (ROS) from epithelial, vascular endothelial, fibroblasts, and tissue macrophages with cycles of amplifications. Within such primary damage response, the DNA damage and ROS lead to three major steps: (1) fibronectin breakdown that activates macrophages ending with stimulation of matrix metalloproteinase; (2) nuclear factor-κB (NF-κB) activation that stimulates the gene expression and release of pro-inflammatory cytokines, e.g., TNF-α, interleukin (IL)-1β, and IL-6; and (3) ceramide pathway through sphingomyelinase and ceramide synthase. The result will be more tissue injury and stimulated apoptosis. During the signal amplification phase, there is restimulation of tissue damage and apoptosis by the major pro-inflammatory cytokines (TNF-α, IL-1β, and IL-6), NF-κB-mediated gene expression, and ceramide and caspase pathways. During the ulceration and loss of the protective barrier, secondary infection adds more pro-inflammatory reactions and complicates the already existing inflammation before the healing phase starts by matrix signaling to basal epithelial cells to migrate, proliferate, and differentiate. Republished with the permission of Dr. Redding. **(A)** Initial phase, **(B)** primary damage phase, **(C)** signal amplification phase, and **(D)** ulcerative phase.

**Figure 3:**
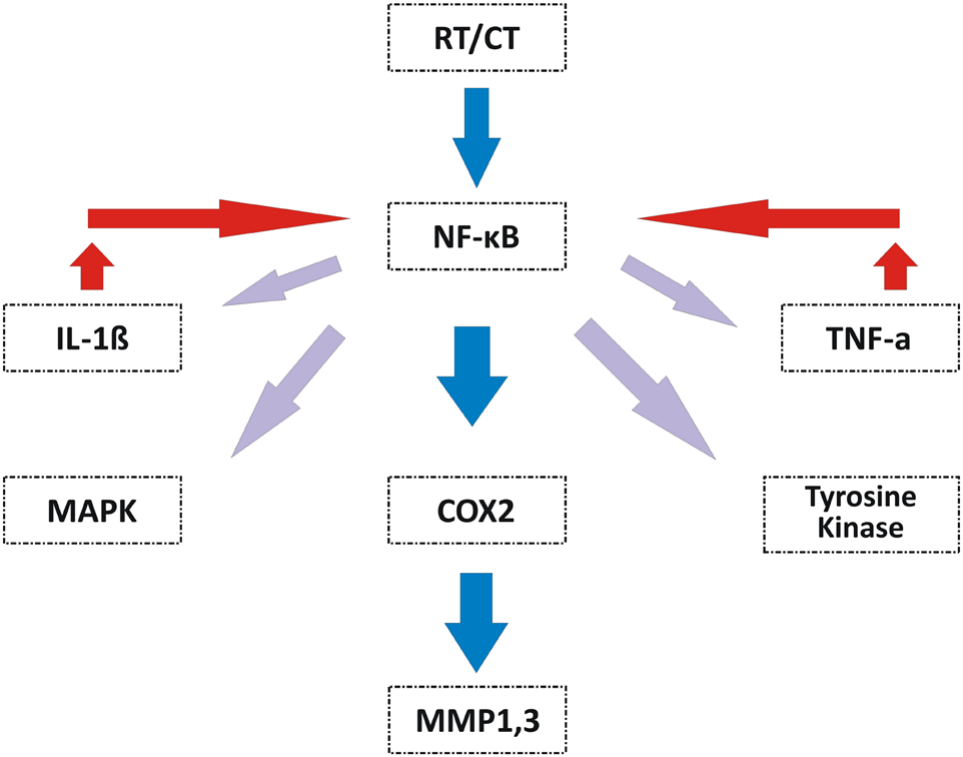
**Signal amplification during OM induced by RT and/or CT (10)**. Signal amplification during RT- and/or CT-induced OM is mediated by activation of NF-κB that is reactivated by IL-1β. NF-κB induces the expression of genes responsible for the MAPK, COX-2, and tyrosine kinase pathways to finally activate the MMP1 and MMP3 signaling at the injured tissue cells. TNF-α, tumor necrosis factor-α; IL-1β, interleukin-1β; NF-κB, nuclear factor-κB; MAPK, mitogen-activated protein kinase; COX-2, cyclooxegenase-2; MMP1, matrix metalloproteinase 1; MMP3, matrix metalloproteinase 3; OM, oral mucositis; CT, chemotherapy. Republished with the permission of Dr. Sonis.

In 2009, Redding summarized Sonis’ RIOM pathobiology phases (Figure 2). The initiation phase with RT and/or CT injury results in direct and lethal DNA damage with the release of reactive oxygen species (ROS) from epithelial and vascular endothelial cells, fibroblasts, and tissue macrophages. This is followed by amplification of this signal (11). During the primary damage response, the DNA damage and ROS act through three major pathways: (1) fibronectin breakdown, which stimulates the macrophages leading to activation of the matrix metalloproteinases (MMPs), (2) nuclear factor-κB (NF-κB) activation, which stimulates the gene expression and the release of pro-inflammatory cytokines, e.g., TNF-α, IL-1β, and IL-6, and (3) ceramide pathway through sphingomyelinase and ceramide synthase. The end result will be more tissue injury and stimulated apoptosis (11). During the signal amplification phase, there is restimulation of tissue damage and apoptosis by the major pro-inflammatory cytokines (TNF-α, IL-1β, and IL-6), NF-κB-mediated gene expression, and ceramide and caspase pathways. The basement membrane protective barrier is lost during the ulceration phase. This leads to Gram-negative and yeast secondary infection potential, which adds more pro-inflammatory reactions and complicates the already existing inflammation. The healing phase starts by matrix signaling to basal epithelial cells to migrate, proliferate, and differentiate (11). Signal amplification during RIOM or CT-induced OM is a main step in this treatment-induced injury, according to Sonis et al. (17). RT and/or CT activate the transcription factor NF-κB in epithelial, endothelial, and mesenchymal cells and macrophages, resulting in upregulation of genes and production of pro-inflammatory cytokines: tumor necrosis factor-α (TNF-α) and IL-1β, which amplify the primary signal and activate NF-κB. This leads to transcription of genes responsible for MAPK, cyclooxegenase-2 (COX-2), and tyrosine kinase signaling molecules. These signaling pathways activate MMPs 1 and 3 in the epithelial and lamina propria cells, which collectively cause tissue injury (10) (Figure 3).

## RIOM Grading and Scoring Scales

There has been more than one grading scale for RIOM. Table 4 shows the comparison of different RIOM scoring scales (14, 21–23).

**Table 4:** **Comparison of OM scoring scales (14, 21–23)**.

Grade	0	1	2	3	4
WHO	None	Soreness ± erythema	Erythema, ulcers, and patient can swallow solid food	Ulcers with extensive erythema and patient cannot swallow solid food	mucositis to the extent that alimentation is not possible
RTOG	None	Erythema of the mucosa	Patchy reaction <1.5 cm, non-contiguous	Confluent reaction >1.5 cm, contiguous	Necrosis or deep ulceration, ±bleeding
WCCNR	Lesions: none	Lesions: 1–4	Lesions: >4	Lesions: coalescing	N/A
	Color: pink	Color: slight red	Color: moderate red	Color: very red	
	Bleeding: none	Bleeding: N/A	Bleeding: spontaneous	Bleeding: spontaneous	

*WHO, World Health Organization; RTOG, Radiation Therapy Oncology Group; WCCNR, Western Consortium for Cancer Nursing Research; OM, oral mucositis*.

**Figure 4:**
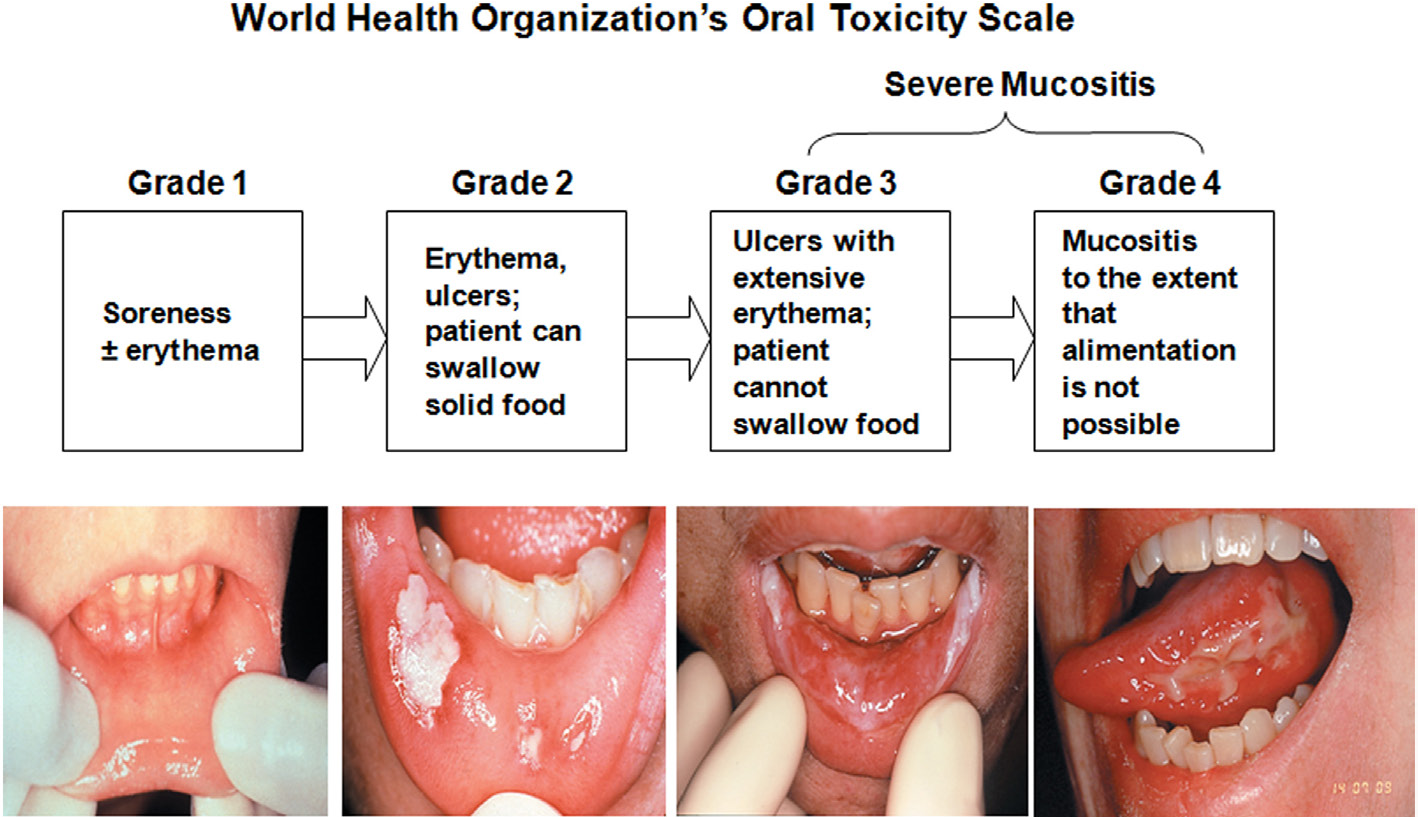
**World Health Organization’s Oral Toxicity Scale**. Republished with the permission of Dr. Patrick Stiff, Loyola University Medical Center, Maywood, IL, USA.

World Health Organization Oral Toxicity Scale measures the anatomical, symptomatic, and functional elements of OM (Figure 4). The Radiation Therapy Oncology Group (RTOG) determined the acute radiation morbidity scoring criteria for mucous membranes. Finally, the Western Consortium for Cancer Nursing Research describes only the anatomical changes associated with OM (24).

Radiation Therapy Oncology Group developed the Acute Radiation Morbidity Scoring Criteria for the evaluation of RT effects (another criterion was generated for late effects of RT) (25). The National Cancer Institute (NCI) Common Toxicity Criteria (NCI-CTC) scores CT-related side effects. The RTOG was gathered with the NCI-CTC to produce version 2.0, which has been used in all NCI clinical trials since March 1998 (Table 5) (2, 25, 26).

**Table 5:** **Toxicity grading of oral mucositis (OM) according to World Health Organization (WHO) and National Cancer Institute Common Toxicity Criteria (NCI-CTC) criteria (2)^a^**.

Side effect	Grade 0 (none)	Grade 1 (mild)	Grade 2 (moderate)	Grade 3 (severe)	Grade 4 (life threatening)
WHO oral mucositis (stomatitis)	None	Oral soreness, erythema	Oral erythema, ulcers, can eat solids	Oral ulcers, requires liquid diet only	Oral alimentation not possible

NCI-CTC chemotherapy-induced stomatitis/pharyngitis (oral/pharyngeal mucositis)	None	Painless ulcers, erythema, or mild soreness in the absence of lesions	Painful erythema, edema, or ulcers, but can eat or swallow	Painful erythema, edema, or ulcers requiring intravenous hydration	Severe ulceration or requires parenteral or enteral nutritional support or prophylactic intubation

NCI-CTC mucositis due to radiation	None	Erythema of the mucosa	Patchy pseudomembranous reaction (patches generally ≤1.5 cm in diameter and non-contiguous)	Confluent pseudomembranous reaction (contiguous patches generally >1.5 cm in diameter)	Necrosis or deep ulceration; may include bleeding not induced by minor trauma or abrasion

NCI-CTC stomatitis/pharyngitis (oral/pharyngeal mucositis) for bone marrow transplantation studies	None	Painless ulcers, erythema, or mild soreness in the absence of lesions	Painful erythema, edema, or ulcers, but can swallow	Painful erythema, edema, or ulcers preventing swallowing or requiring hydration or parenteral (or enteral) nutritional support	Severe ulceration requiring prophylactic intubation or resulting in documented aspiration pneumonia

*^a^Republished with the permission of Dr. Christoph C. Zielinski*.

The OM Index (OMI) scores the severity of OM by the erythema, ulceration, atrophy, and edema (a scale of 0–3 was designated for each element: 0 = none and 3 = severe). The OMI is considered internally consistent with high test–retest and interscorer reliability, and it shows solid validity (27).

The OM Assessment Scale (OMAS) is a highly reproducible scoring scale for RIOM, responsive over time, and accurate in detecting OM-associated elements (25). OMAS records the objective assessment of OM depending on the scoring of the presence and size of the ulceration or pseudomembrane (score 0–3: 0 = no lesion; 1 = lesion of <1 cm^2^; 2 = lesion of 1–3 cm^2^; 3 = lesion >3 cm^2^) and erythema (score 0–2: 0 = none; 1 = not severe; 2 = severe) on the upper and lower lips, right and left cheeks, right and left ventral and lateral tongue, floor of the mouth, soft palate, and hard palate (23, 28).

All these scoring scales are validated and are required in assessing RIOM and the therapeutic benefits of any new treatment of RIOM.

## Diagnosis of RIOM

Radiation-induced oral mucositis can develop within or after 2 weeks from the beginning of RT. Oral assessment guide could be a useful tool for detection of early OM (Table 6) (20). Apart from the early clinical signs and symptoms, CBC with differential is considered the baseline to help radiation oncologists to determine the most susceptible time for developing OM or oral infection. Radiation oncologists can start the RT provided that there is no evidence of any periodontal disease. If at any point RIOM develops, oral lesion culture and antimicrobial therapy are recommended as soon as possible (29). Since renal diseases are considered contributing factors for OM (15), chemistry levels should be regularly monitored by the treating physician (29).

**Table 6:** **Oral assessment guide (30)**.

Item/grade	1	2	3
Voice	Normal	Deeper or raspy	Difficulty talking
Swallow	Normal	Some pain	Unable to swallow
Lips	Smooth pink and moist	Dry or cracked	Ulcerated or bleeding
Tongue	Pink and moist	Coated and shiny ± red	Blistered or cracked
Saliva	Watery	Thick	Absent
Mucus membrane	Pink and moist	Red and coated without ulcers	Ulcers
Gingiva	Pink and firm	Edematous ± redness	Spontaneous or pressure-induced bleeding
Teeth/denture areas	Clean, no debris	Plaque and localized debris	Generalized plaque or debris

## Differential Diagnosis of RIOM

**Figure 5:**
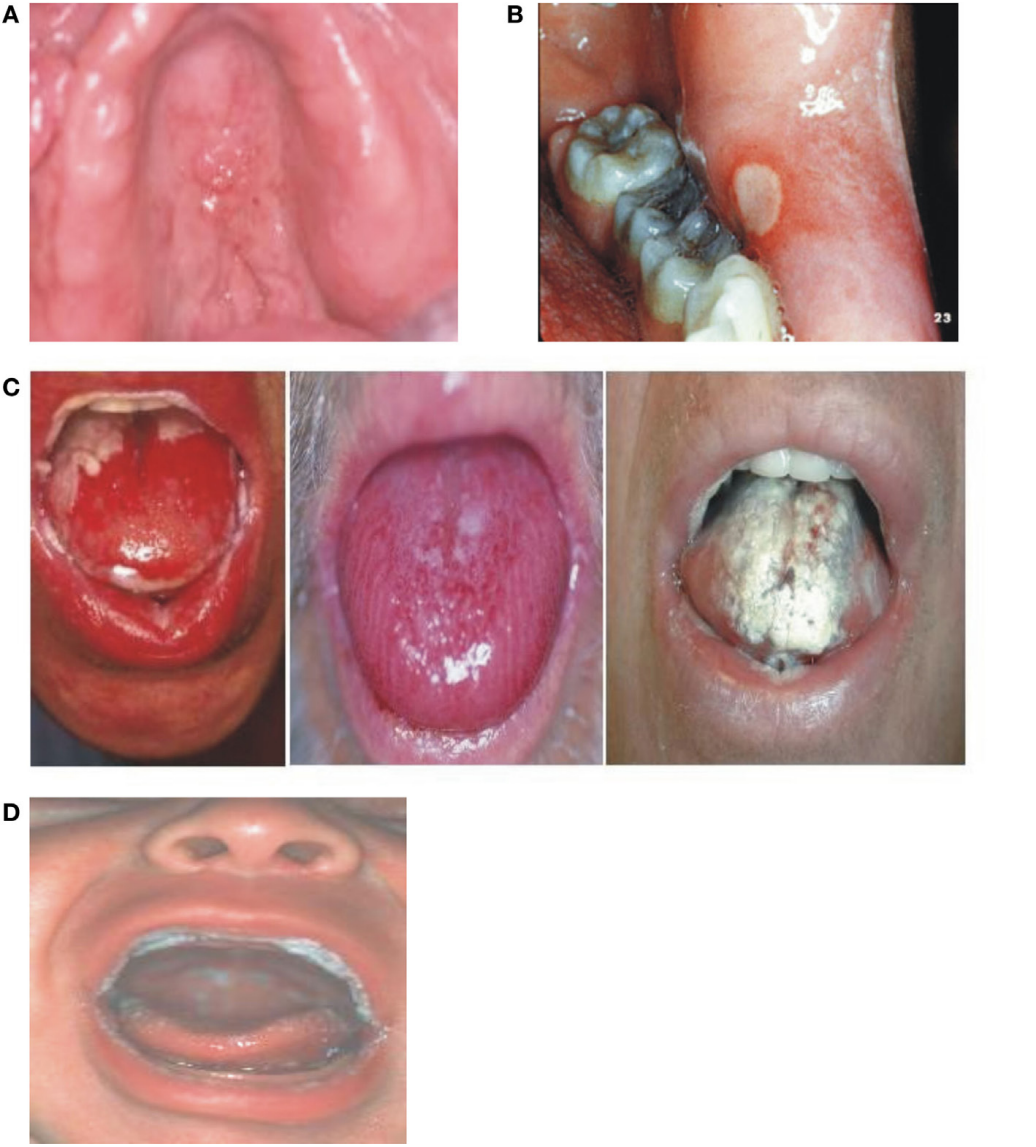
**Differential diagnosis of radiation-induced oral mucositis**. Republished with the permission of Dr. Patrick Stiff, Loyola University Medical Center, Maywood, IL, USA. **(A)** Local, denture-related lesion, **(B)** aphthous ulcer, **(C)** oral mucositis, and **(D)** oral thrush.

Because similar conditions can coexist in immunocompromised patients including cancer patients receiving RT and/or CT, differential diagnosis for RIOM is critical. Table 7 shows possible similar conditions (Figure 5) (20, 31).

**Table 7:** **Differential diagnosis of RIOM (20, 31)**.

Disease/injury	Cause	Clinical presentation/lab findings	Severity	Treatment options
Oral mucositis	Chemotherapy and radiation therapy	Diffuse redness, ulcerations, and pain, particularly in areas where teeth abut tissue	Varies; in BMT setting up to 98% have grade 3/4	Palliative rinses, narcotics, palifermin in the BMT setting
Aphthous stomatitis	Etiology not identified	Single painful ulcer	Localized, but painful; maximum grade 2	Topical
Herpetic mucositis	HSV1	Usually several spots; ulcerative	Usually grade 1–2	Acyclovir, valacyclovir, foscarnet
Oral thrush	Candida	Varies from painless to mild soreness; whitish plaques	Usually grade 0–1	Nystatin rinses; fluconazole and other azoles
Denture/oral trauma	Dentures	Common in elderly patients with loose-fitting dentures	Can limit calories	Repair, removal of dentures
Gangrenous stomatitis	Bacterial infections	Necrotic pseudomembranes	Rare, can be severe	Antibacterials that treat oral aerobes and anaerobes
Acute necrotizing stomatitis	Bacterial infections in immune-deficient patients	Pain, fever, necrotic, bloody ulcers	Grade 3/4	Control of infection

*BMT, bone marrow transplantation; RIOM, radiation-induced oral mucositis; HSV1, herpes simplex virus type 1*.

## Prognosis of RIOM

The general long-term prognosis is reasonably good since most lesions resolve within 2–4 weeks after stopping the RT or CT. Although RIOM is considered a self-limited injury in some patients, it could be a lethal injury in moderately to severely ill patients, which could lead to ICU admission with obligatory cessation of RT. Patient losses are a common event under these circumstances (32).

## Prevention of RIOM

Maintaining good oral care is the main preventive measure for RIOM to minimize the risk for candidiasis or secondary bacterial infection, especially in hyperfractionated radiotherapy, combined CCRT regimens, or RT combined with a targeted agent due to increased mucositis severity (3). We will summarize the most recent agents and measures to prevent RIOM.

1. Good oral hygiene
  Good oral hygiene has been found to be one of the most effective ways to lower the risk of RIOM and minimize its progression. Pre-existing oral pathology, e.g., dental caries, periodontal lesions, pulpal disease, and oral xerostomia, has been linked with increased bacteria colonization and severe RIOM. It is recommended to do early oral examination before starting any mucosal toxic therapy for cancer patients. To help minimize the oral side effects of antineoplastic therapy, it is recommended to eliminate any oral pathology before the beginning of RT. This may be accomplished by performing early histological, cytological, microbiologic, and serologic examinations (2). The Multinational Association of Supportive Care in Cancer (MASCC) and the International Society of Oral Oncology (ISOO) guidelines recommend the use of a standardized oral care protocol, e.g. brushing with a soft toothbrush, flossing and the use of non-medicated rinses (saline or sodium bicarbonate rinses) (Table 9) (33–36). The good oral care can be summarized as follows:
  – Rinsing with a non-irritating solution, e.g., saline to increase the quality of saliva.
  – Daily ultrasoft tooth brushing with fluoride toothpaste.
  – Scaling and cleaning.
  – Very soft diet with low sugar and non-acidic food and drinks (Table 8).
  – Flossing is not recommended due to low platelet count.
  – Minimize denture use.
  – No smoking or alcohol.
  – Other preventive procedures include minimizing the microbial load (will be discussed more in the treatment section) and educating the patient on good oral hygiene, which is mandatory.
2. Cryotherapy has been recommended for CT-induced OM, but no proven role in RIOM due to insufficient evidence (33).
3. Keratinocyte growth factor is an epithelial mitogen that reduces the levels of ROS by activating nuclear factor (erythroid-derived 2)-like 2 and had been used in RIOM with promising results (37–53). It appears to be one of the promising treatment and prevention options for RIOM that has been investigated in clinical trials (39, 43). Palifermin (IV recombinant human KGF-1) had been approved by the US-FDA for minimizing OM in hematologic malignancies’ patients who receive myelotoxic therapies and require hematopoietic cell support after its reliable results in alleviating WHO grade 3 and 4 OM in these patients. Palifermin is delivered IV 3 days before of CT/RT and for 3 days after CT. Palifermin should be avoided on the same day of CT/RT (33).
4. Amifostine is a free-radical scavenger, antioxidant, and cytoprotective agent that was administered subcutaneously (SC) and IV in many clinical trials for RIOM. Amifostine is conventionally given IV before RT or CT. It is approved by the US-FDA to reduce the cumulative renal toxicity associated with repeated administration of cisplatin in patients with advanced ovarian cancer. In addition, it was approved for by the US-FDA to reduce the incidence of moderate to severe xerostomia in patients undergoing postoperative RT for head and neck cancer, where the radiation port includes a substantial portion of the parotid glands (33, 54–62). Although there was a reduction in the pro-inflammatory cytokine production, its side effects, e.g., hypotension and nausea, were recorded, particularly with IV route. Nevertheless, SC injection 60 min before RT in head and neck cancer patients showed marked reduction of these side effects, unfortunately, with reduced efficacy and patient compliance. Only cutaneous toxicity was noted in SC route of amifostine delivery (54, 55). For moderate to severe RT-induced xerostomia in head and neck cancer patients, the recommended dose of amifostine is 200 mg/m^2^ once daily over 3 min IV, starting 15–30 min before standard fraction RT (1.8–2.0 Gy). Blood pressure should be monitored before, during, and after the IV infusion. Oral 5-HT3 receptor antagonists with/without other antiemetics are recommended before amifostine therapy (63–65).
5. Radiation shields (intraoral devices), midline mucosa-sparing blocks, 3-D and RT field design, intensity-modulated radiation therapy (IMRT), and removal of separable prosthetics are shown in preclinical studies to reduce the radiation scatter and the RIOM injury (66–69).
6. Low-energy helium–neon laser applied before RT showed significant reduction in the duration and the severity of RIOM in head and neck cancer patients (70). MASCC/ISOO guidelines suggest the use of low-level laser therapy in CT-induced OM at centers that can provide the necessary technology and training (33).

**Table 8:** **Diet recommended for RIOM patients (20)**.

Typically accessed diet	Things to avoid	Habits to avoid
Liquids	Rough food (potato chips, crisps, toast)	Smoking
Purees	Spices	Alcohol
Ice	Salt	
Custards	Acidic fruit (grapefruit, lemon, orange)	
Non-acidic fruits (banana, mango, melon, peach)		
Soft cheeses		
Eggs		

**Table 9:** **Multinational Association for Supportive Care in Cancer/International Society for Oral Oncology (MASCC/ISOO) Clinical Practice Guidelines for oral mucositis (3)**.

Intervention/mode of administration	Purpose	Cancer treatment	Level of evidence
**Recommendations in favor of an intervention (strong evidence supports effectiveness in the treatment setting listed)**			

Oral cryotherapy for 30 min	Prevention of OM	Patients receiving bolus 5-fluorouracil chemotherapy	Level II

Recombinant human keratinocyte growth factor-1 (palifermin) at a dose of 60 µg/kg per day for 3 days prior to conditioning treatment and for 3 days after transplant	Prevention of OM	Patients receiving high-dose chemotherapy and TBI, followed by autologous stem cell transplantation, for a hematological malignancy	Level II

Low-level laser therapy (wavelength at 650 nm, power of 40 mW, and each square centimeter treated with the required time to a tissue energy dose of 2 J/cm^2^)	Prevention of OM	Patients receiving HSCT conditioned with high-dose chemotherapy, with or without TBI	Level II

Patient-controlled analgesia with morphine	Pain reduction	Patients undergoing HSCT	Level II

Benzydamine mouthwash	Prevention of OM	Patients with HNC receiving moderate dose radiation therapy (up to 50 Gy), without concomitant chemotherapy	Level II

**Suggestions in favor of an intervention (weaker evidence supports effectiveness in the treatment setting listed)**			

Oral care protocols	Prevention of OM	All age groups and across all cancer treatment modalities	Level III

Oral cryotherapy	Prevention of OM	Patients receiving high-dose melphalan, with or without TBI, as conditioning for HSCT	Level III

Low-level laser therapy (wavelength around 632.8 nm)	Prevention of OM	Patients undergoing radiotherapy, without concomitant chemotherapy, for HNC	Level III

Transdermal fentanyl	Pain reduction	Patients receiving conventional or high-dose chemotherapy, with or without TBI	Level III

2% morphine mouthwash	Pain reduction	Patients receiving chemoradiation for HNC	Level III

0.5% doxepin mouthwash	Pain reduction	All patients with OM-induced pain	Level IV

Systemic zinc supplements administered orally	Prevention of OM	HNC patients receiving radiation therapy or chemoradiation	Level III

**Recommendations against interventions (strong evidence indicates lack of effectiveness in the treatment setting listed)**			

PTA (polymyxin, tobramycin, amphotericin B) and BCoG (bacitracin, clotrimazole, gentamicin)	Prevention of OM	Patients receiving radiation therapy for HNC	Level II

Iseganan antimicrobial mouthwash	Prevention of OM	Patients receiving high-dose chemotherapy, with or without TBI, for HSCT or in patients receiving radiation therapy or concomitant chemoradiation for HNC	Level II

Iseganan antimicrobial mouthwash	Prevention of OM	Patients receiving high-dose chemotherapy, with or without TBI, for HSCT or in patients receiving radiation therapy or concomitant chemoradiation for HNC	Level II

Sucralfate mouthwash	Prevention of OM	Patients receiving chemotherapy for cancer (I), or inpatients receiving radiation therapy (I) or concomitant chemoradiation (II) for HNC	Level I, II

Sucralfate mouthwash	Treatment of OM	Patients receiving chemotherapy for cancer (I), or in patients receiving radiation therapy (II) for HNC	Level I, II

Intravenous glutamine	Prevention of OM	Patients receiving high-dose chemotherapy, with or without TBI, for HSCT	Level II

**Suggestions against interventions (weaker evidence indicates lack of effectiveness in the treatment setting listed)**			

Chlorhexidine mouthwash	Prevention of OM	Patients receiving radiation therapy for HNC	Level III

Granulocyte-macrophage colony-stimulating factor mouthwash	Prevention of OM	Patients receiving high-dose chemotherapy, for autologous or allogeneic HSCT	Level II

Misoprostol mouthwash	Prevention of OM	Patients receiving radiation therapy for HNC	Level III

Systemic pentoxifylline, administered orally	Prevention of OM	Patients undergoing HSCT	Level III

Systemic pilocarpine, administered orally	Prevention of OM	Patients receiving radiation therapy for head and neck cancer (III), or patients receiving high-dose chemotherapy, with or without TBI, for HSCT (II)	Level II and III

*OM, oral mucositis; HSCT, hematopoietic stem cell transplantation; TBI, total body irradiation; HNC, head and neck cancer*.

*Criteria for each level of evidence (34)*.

*Level I: evidence obtained from meta-analysis of multiple, well-designed, controlled studies; randomized trials with low false-positive and false-negative errors (high power)*.

*Level II: evidence obtained from at least one well-designed experimental study; randomized trials with high false-positive and/or false-negative errors (low power)*.

*Level III: evidence obtained from well-designed, quasi-experimental studies such as non-randomized, controlled single-group, pretest–posttest comparison, cohort, time, or matched case–control series*.

*Level IV: evidence obtained from well-designed, non-experimental studies, such as comparative and correlational descriptive and case studies*.

*Level V: evidence obtained from case reports and clinical examples*.

## Symptomatic Treatment of RIOM

No single agent has been approved by the US-FDA for the treatment of RIOM. Symptoms reduction and complications prevention of RIOM, including nutritional support, pain control, prophylaxis, and/or treatment of secondary infections, are considered the main cornerstone in the management of RIOM (34–36). Agents that were investigated and/or applied in RIOM treatment are discussed in the context of recently updated evidence-based preclinical and clinical studies.

- Locally applied agents
  1. Glycyrrhetinic acid/povidone/sodium hyaluronate gel has mechanical action implemented in the relief of pain in RIOM. It adheres to the mucosal surface of the mouth, soothing oral lesions. Nevertheless, the preclinical studies are controversial, and only one clinical trial on unknown results was conducted to date (71).
  2. l-Glutamine is a non-essential amino acid that counteracts RT-induced metabolic deficiencies (72). Locally applied l-glutamine reduced the RIOM in a randomized clinical trial (73). Glutamine powder for oral suspension was approved by the US-FDA for topical application in management of CT-induced OM, mainly IOMyet (74).
  3. Manganese superoxide dismutase is a detoxifying agent that removes ROS. It was shown to have radioprotective effects against RT-induced colitis, esophagitis, hepatic cells apoptosis, and intestinal and eye injury (75–98). Phase I dose escalation study of GC4419 (manganese-containing macrocyclic ligand complex similar to naturally occurring superoxide dismutase enzymes) in combination with CT/RT for squamous cell cancer of the head and neck has just been completed waiting for results release (NCT01921426).
  4. Local anesthetics, e.g., diphenhydramine, viscous xylocaine, lidocaine, and dyclonine hydrochloride, are used for short-term relief of pain associated with RIOM, despite the fact that they can interfere with the taste sensation leading to hypoalimentation (99, 100). Swishing and gargling the anesthetic viscous gel containing 2% lidocaine and holding 5 mL of it in mouth for 1 min then spitting it out before meals have been shown to be helpful for better alimentation (101). One clinical trial showed dyclonine hydrochloride to have a superior effect among all other agents without significant difference recorded (102). The most effective anesthetic agent is still to be determined. Benzocaine gel is another locally applied bioadhesive agent containing benzyl alcohol (10%) and is used to relieve pain and facilitate eating and drinking in mild and moderate RIOM (103). Benzocaine-containing lozenges are diluted to alleviate the pain sensation and mechanical sensitivity in mild to moderate OM (104, 105). The “magic mouthwash” (lidocaine, diphenhydramine, magnesium aluminum hydroxide) and morphine mouth washes are preferable and have been reported by patients to be effective in alleviating pain in RIOM (106–108).
  5. The application of corticosteroids mouthwashes has shown promising results. The limited availability of a large-scale data is a gap that should be bridged through relevant clinical studies (109).
  6. Allopurinol and uridine were shown to be effective in reducing 5-fluorouracil oral toxicity in preclinical studies (110–114). Despite these results, they were ineffective approaches in randomized clinical trials as a therapy to reduce the treatment-related oral toxicity (115, 116).
  7. Chlorhexidine is a bisguanidine exhibiting broad-spectrum antibacterial and antimycotic activities. The clinical trials done with chlorhexidine concluded that it cannot be recommended for the prophylaxis or the treatment of RIOM (117–120). Alcohol-containing chlorhexidine mouth rinse should be avoided during clinical oral ulceration. Therefore, the MASCC/ISOO guidelines recommend against the use of chlorhexidine mouth rinse for prevention or treatment OM (33).
  8. Artificial saliva spray is an over-the-counter agent frequently used to alleviate mucosal dryness in mild cases of RIOM (121).
  9. Chamomile has anti-inflammatory, antipeptic, antispasmodic, and antibacterial effects. It was investigated with encouraging results as an emulsion therapy for CT-induced mucositis (122–126). Studies are needed for its application in RIOM to determine its efficacy.
  10. Honey has been investigated in many preclinical studies due to its mucosal protective effect that was confirmed as a reduction in the incidence and severity of RIOM (127–132). However, the available clinical trial used only Manuka honey, and it appears to contradict the preclinical studies’ results (133). More studies are needed to confirm the therapeutic potential of honey in RIOM.
  11. Sucralfate is a basic aluminum salt of sucrose sulfate that was used as mouthwash to reduce the intensity of RIOM and CCRT-induced mucositis as well (39, 100, 134–151). Despite its long application history, it is considered to have little effect in RIOM when compared to oral hygiene and symptomatic mucositis therapy (2). MASCC/ISOO Mucositis Guidelines did not find enough evidence for the beneficial application of sucralfate in OM (34).
  12. Vitamin A and its derivatives have anti-inflammatory and epithelial proliferative effect (152). Topical tretinoin has been shown to reduce the oral complications during bone marrow (BM) transplantation (153).
  13. Vitamin-E (tocopherol) has been shown to lower the oxidative damage of the oral mucosa and reduce the incidence of symptomatic RIOM in head and neck cancer patients in a randomized double-blind clinical trial (152, 154).
  14. Sodium alginate was shown to reduce the discomfort and the severity of RIOM in a randomized clinical trial (155).
  15. Benzydamine hydrochloride is a non-steroidal antimicrobial, anti-inflammatory, anesthetic, and analgesic agent that reduces pro-inflammatory cytokine production, scavenges the ROS, and acts as membrane stabilization and as an antimicrobial agent (118, 156, 157). When compared with chlorhexidine, patients with RIOM treated with benzydamine hydrochloride found more discomfort (118). Benzydamine hydrochloride failed to be approved by the US-FDA for OM management. Because of a negative interim analysis, a recent phase III trial for benzydamine hydrochloride therapy in RIOM was stopped (33).
  16. Povidone-iodine is an antiviral, antibacterial, and antifungal agent. Randomized clinical study showed that povidone-iodine reduces the incidence, severity, and duration of CCRT-induced OM, in addition to its advantages of being cheap and easily applied (118, 158–160).
  17. Capsaicin is an inhibitor of neutrophils that reduces the pain sensation. One clinical trial showed that orally applied capsaicin caused temporary relief of pain in mucositis caused by RT and CT (161). However, more studies are needed for optimization of its analgesic effect.
- Systemically applied agents
  1. Cyclooxegenase-2 inhibitors that have different mechanisms of action were applied in the management of RIOM. They suppress NF-κB, reduce pro-inflammatory-cytokine production, and inhibit angiogenesis (162–164). A randomized placebo-controlled trial showed that prophylactic systemic administration of indomethacin, a COX-2 inhibitor, significantly lowered the severity and delayed the onset of RIOM (165). In addition, PG E1 and E2 showed improvement in OM induced by RT and CT in few studies; however, their application is still controversial (166–172).
  2. *N*-acetylcysteine is an antioxidant that has been shown to suppress NF-κB activation (173, 174). Because of its proven radioprotective role in RT-induced dermatitis, bone injury, liver toxicity, and intestinal injury (173–188), *N*-acetylcysteine was recommended as a candidate for a trial in RIOM. In a placebo-controlled phase II trial of patients with head and neck cancer, *N*-acetylcysteine significantly reduced the severity of RIOM (33).
  3. Colony-stimulating factor and granulocyte-macrophage colony-stimulating factor (GM-CSF) systemic therapy recruit neutrophils to the tissue injury site (189). Local application of GM-CSF mouthwash was shown marked alleviation of RIOM in several studies (2). Nevertheless, in clinical trials, its systemic application therapeutic value appears controversial (190, 191). SC GM-CSF reduced the severity of OM in patients treated with accelerated RT (192). In another randomized clinical study, systemic GM-CSF reduced the incidence of RIOM; however, another study did not show the same result (140, 193). Systemic GM-CSF therapeutic potential is still controversial and requires further investigation.
  4. Transforming growth factor-β3 inhibits the oral basal cell proliferation. It was shown to reduce the incidence of CT-induced mucositis (194). However, a reliable clinical trial is needed to assess its therapeutic potential with RT.
  5. Beta-carotene’s antioxidative effect (195, 196) was implemented in a randomized clinical trial where there was a significant reduction in the incidence of severe OM in CCRT (197).
  6. Analgesics are strong candidates for alleviating the pain related to RIOM. A retrospective study showed that opioid therapy remains a corner stone for OM pain management in CCRT, as suggested by the MASCC/ISOO guidelines (33, 198).
  7. Azelastine is a potent second-generation selective histamine antagonist that is used as an anti-inflammatory and antioxidant agent. One clinical trial showed significant reduction in the incidence and the severity of OM with CCRT (199).
  8. Propantheline is an anticholinergic agent that reduces the salivary flow. One clinical trial showed that propantheline and oral cryotherapy may be feasible and effective in reducing mucosal toxicity in cancer patients receiving high-dose CT (200). However, studies are needed for RIOM.
  9. Immunoglobulins have lower salivary and systemic levels in patient receiving antineoplastic therapy. They have immune-modulating and anti-inflammatory properties. Intravenous or intramuscular immunoglobulins are frequently applied as prophylactic and therapeutic options for RIOM (158, 201).
  10. Systemic corticosteroids were used in RIOM management. A double-blind placebo-controlled randomized trial has shown a tendency toward reduced RT interruption in prednisone-treated relative to placebo-treated patient groups without evidence of reduced RIOM incidence or severity (202).
  11. Pentoxifylline regulates endotoxin-induced production of TNF-α. Although the preclinical studies showed significant reduction in the severity of RIOM with pentoxifylline (203), the clinical trials show that it is not effective in reducing the antineoplastic oral toxicity (204–208).
  12. Salicylic acid derivatives should be avoided due to the increased risk for bleeding (34–36).
  13. Sphingomyelinase and ceramide synthase inhibitors can be a potential candidate for RIOM. They inhibit the ceramide pathway-mediated RT-induced apoptosis (209–216). No current clinical trials have been started for them yet.
- Oral microbial load reduction agents
  1. Antimicrobial agents showed beneficial effect in prophylaxis and reduction of the severity of RIOM. RT injury leads to a change in the mucosal membrane barrier, salivary flow, and composition which favor the growth and colonization of different bacterial species, mainly Gram-negative bacteria. Many preclinical studies have investigated the therapeutic effect of different antimicrobial agents in RIOM (217–220). The FDA has granted fast track designation for brilacidin-OM, an oral rinse formulation of defensin-mimetic brilacidin (221–223), for the prevention of OM. There is a current phase II clinical trial to evaluate the safety and efficacy of brilacidin oral rinse in patients with head and neck cancer (NCT02324335).
  2. Fungal infections are not involved directly in the development in RIOM, rather they can complicate the situation, especially in immunocompromised patients, and that is why the use of antifungal agents have been applied in RIOM treatment. A clinical study has shown that systemic fluconazole prophylaxis caused a significant beneficial effect on the severity of OM and on radiotherapy interruptions (224). The same effect was noted in randomized clinical trials investigating clotrimazole (2). Some oral mouthwashes containing amphotericin B have shown similar effects; however, due to carrier allergy, there might be a limitation in its application (225).
  3. Antibacterial agents have been investigated in mucositis depending on a hypothesis stating that aerobic species (e.g., *Pseudomonas* spp. and *Staphylococcus epidermidis*), anaerobic bacteria (e.g., *Bacteroides* spp., and *Veillonella* spp.), and endotoxin of aerobic Gram-negative bacilli are considered a main contributor in the development of the secondary infection phase in RIOM (2, 226). Antibiotic lozenges with polymyxin-E and tobramycin have protected against severe mucositis when compared to placebo or chlorhexidine (227). In addition, ciprofloxacin- and ampicillin-containing mouthwashes showed similar effect (228, 229).
  4. Antiviral agents against herpes simplex virus (HSV) type I and varicella zoster virus (VZV) were applied topically and systematically. HSV and VZV are the most common viral infections that aggravate RIOM in seropositive and myelo-suppressed patients (230–232). Systemic and topical acyclovir was investigated and applied in RIOM management and caused a reduction in the oral herpetic infections without an evident prophylactic role against OM itself (233–238).

## Cellular Therapies for RIOM

Bone marrow-derived mesenchymal stromal cells (bmMSCs) therapy have been applied in fractionated radiation-induced OM where the administration of a systemic single dose of six million MSCs resulted in a significant decrease in ED_50_ (the RT dose that produces ulcer in 50% of irradiated mice) (239). The first MSCs therapy for RIOM was done in 2014 by Schmidt et al. (239). They concluded that transplantation of BM or bmMSCs could modulate RIOM in fractionated RT, depending on the time of transplantation (239). Nevertheless, in another study, the authors concluded that bmMSCs transplantation had no therapeutic benefits on RIOM in single-dose RT when compared to the therapeutic effect of mobilization of endogenous BM stem cells (240). More studies are needed in this field building on the initial studies, which showed significant and clinically relevant therapeutic gain of MSCs therapy for RIOM (Table 10).

**Table 10:** **Radiation-induced oral mucositis (RIOM) the clinical trials that have been done until 2001 (2)^a^**.

Injury	Reference	Randomized/controlled/double blind	P/T	Application/doses	Results
RT	Shieh et al. (241)	Yes/yes/no	P	Instructions on oral care	Significant reduction

RT	Perch et al. (68)	No/no/no	P	Midline mucosa-sparing blocks	Decreased mucositis without affecting tumor control

RT	Rugg et al. (242)	No/no/no	P	Smoking during RT	Higher mucositis incidence in smokers

RT	Scherlacher et al. (243)	Yes/yes/no	P	Sucralfate vs. standard oral hygiene	Significant reduction of incidence and severity of mucositis

RT	Allison et al. (146)	Yes/yes/no	P + T	Sucralfate + fluconazole vs. standard oral care	Significant reduced severity and symptomatic relief

RT	Franzen et al. (145)	Yes/yes/yes	P	Sucralfate vs. placebo	Significant lower incidence of severe mucositis

RT	Makkonen et al. (147)	Yes/yes/yes	P	Sucralfate vs. placebo	Only slight protective effect of sucralfate

RT	Epstein et al. (148)	Yes/yes/yes	P + T	Sucralfate vs. placebo	Non-significant reduction of oral discomfort

RT	Meredith et al. (144)	Yes/yes/yes	T	Antacid, diphenhydramine, lidocaine ± sucralfate	Non-significant reduction of severity

RT	Cengiz et al. (142)	Yes/yes/yes	P + T	Sucralfate vs. placebo	Decreased severity

RT	Carter et al. (244)	Yes/yes/yes	P	Sucralfate vs. placebo	No difference

RT	Barker et al. (100)	Yes/yes/yes	P + T	Oral hygiene + sucralfate vs. diphenhydramine + kaolin-pectin	No difference

RT	Feber et al. (245)	Yes/yes/no	P	Hydrogen peroxide vs. saline	Significantly more oral discomfort

RT	Spijkervet et al. (246)	Yes/yes/yes	P + T	Chlorhexidine vs. placebo	No difference

RT	Foote et al. (117)	Yes/yes/yes	P + T	Chlorhexidine vs. placebo	Slight aggravation

HD-CT + RT	Ferretti et al. (247)	Yes/yes/yes	P + T	Chlorhexidine vs. placebo	Significant reduction of incidence and severity in the CT group only

CT + RT	Rahn et al. (157)	Yes/yes/no	P	Nystatin, rutosides, immuno-globuines, panthenol ± PVP-iodine	Significant reduction

CT + RT	Adamietz et al. (159)	Yes/yes/no	P	Nystatin, rutosides, immuno-globulines, panthenol ± PVP-iodine	Significant reduction

CT + RT	Hasenau et al. (248)	No/yes/no	P	Hydrogen peroxide, PVP-iodine, dexpanthenol, nystatin	Lower incidence and severity of oral mucositis

RT	Spijkervet et al. (227)	No/yes/no	P	Lozenges of polymyxin, tobramycin, amphotericin vs. historical controls	Lower incidence of mucositis

RT	Mattews et al. (228)	Yes/yes/no	P	Sucralfate + (ciprofloxacin or ampicillin) + clotrimazole vs. sucralfate	Significant reduction of incidence and severity

RT	Symonds et al. (249)	Yes/yes/yes	P	Pastilles containing polymyxin, tobramycin, amphothericin vs. placebo	Significant reduction of severe mucositis

RT	Okuno et al. (250)	Yes/yes/yes	P + T	Lozenges of polymyxin, tobramycin, amphotericin vs. placebo	Significant reduction of oral discomfort, no objective difference

RT	Okuno et al. (250)	Yes/yes/no	T	Amphotericin + colistin + tobramycin + chlorhexidine vs. placebo	Decreased oral discomfort

RT	Symonds et al. (249)	Yes/yes/yes	P	Amphotericin + tobramycin + polymyxin vs. placebo	Significant reduction of the incidence of sever mucositis

RT	Spijkervet et al. (227)	No/yes/no	P	Amphotericin + tobramycin + polymyxin vs. historical chlorhexidine or placebo group	Significant reduction of severity of mucositis

RT	Carl et al. (251)	No/yes/no	P + T	Chamomile vs. historical group	Low incidence of mucositis

RT	Fidler et al. (126)	Yes/yes/yes	P	Chamomile vs. placebo, cryoprophylaxis in all patients	No difference

RT	Abdelaal et al. (252)	No/no/no	P	High-dose betamethasone	Impressive prevention of mucositis incidence

RT	Kim et al. (253)	Yes/yes/yes	P + T	Benzydamine vs. placebo	Significant reduction (less pain)

RT	Epstein et al. (156)	Yes/yes/yes	P + T	Benzydamine vs. placebo	Significant reduction of incidence and severity

RT	Samaranayake et al. (254)	Yes/no/no	P	Benzydamine vs. chlorhexidine	No difference (more discomfort)

CT + RT	Prada et al. (255)	Yes/yes/yes	P + T	Benzydamine vs. placebo	Significant reduction

RT	Huang et al. (73)	Yes/yes/yes	P	Parenteral glutamine vs. placebo	No difference

CT + RT	Porteder et al. (256)	No/yes/no	P	PGE2 or nothing	Significant reduction (less pain)

RT	Matejka et al. (171)	No/yes/no	T	PGE2 tablets four times a day	Reduction of mucositis severity

CT + RT	Hasenau et al. (248)	No/no/no	P + T	P + T hydrogen peroxide, nystatin	Lower incidence of mucositis

RT	Rothwell et al. (109)	Yes/yes/yes	P	Hydrocortisone, nystatin, tetracyclines, diphenhydramine vs. placebo	Significant reduction of incidence

RT	Maciejewski et al. (257)	No/yes/no	P	Applied to one side of buccal mucosa	Significant reduction compared with contralateral side

RT	Barker et al. (100)	Yes/yes/yes		Oral hygiene + sucralfate vs. diphenhydramine + kaolin-pectin	No difference

CT + RT	Berger et al. (161)	No/yes/no	T	Capsaicin in a candy vehicle	Significant temporary pain relief

CT + RT	Mills (197)	Yes/yes/no	P	Beta-carotene or nothing	Decreased severity in the treatment group

RT	Bourhis et al. (258)	Yes/yes/no	P	Amifostine or nothing	Marked reduction of mucositis (tolerance was poor)

RT	Koukourakis et al. (259)	Yes/yes/yes	P	Amifostine vs. saline	Significant reduction of mucositis

RT	Schonekas et al. (260)	No/yes/no	P	Amifostine vs. controls	Significant reduction of mucositis

RT	Wagner et al. (261)	Yes/yes/no	P	Amifostine or nothing	Significant reduction of mucositis

CT + RT	Buntzel et al. (262)	Yes/yes/no	P	Amifostine or nothing	Significant reduction of mucositis and xerostomia

CT + RT	Peters et al. (263)	Yes/yes/no	P	Amifostine or nothing	No significant difference

CT + RT	Vacha et al. (264)	Yes/yes/no	P	Amifostine or nothing	Trend toward reduction of mucositis

CT + RT	Osaki et al. (199)	Yes/yes/no	P	Vitamins C + E, glutathione ± azelastine	Significant reduction

RT	Pillsbury et al. (165)	Yes/yes/yes	P	Indomethacin vs. placebo	Significant delay of mucositis onset

CT + RT	Mose et al. (201)	No/yes/no	P	i.m. immunoglobulins	Significant reduction in CT + RT patients, no difference in RT

RT	Wagner et al. (265)	Yes/yes/no	P	RT + GM-CSF vs. historical control	Significant lower severity of mucositis

RT	Makkonen et al. (140)	No/yes/no	P	Sucralfate ± GM-CSF	No difference

RT	Kannan et al. (193)	No/yes/no	P	RT + GM-CSF	Lower incidence of severe mucositis

CT + RT	Rosso et al. (266)	No/yes/no	P	GM-CSF vs. historical control sig. lower incidence of severe mucositis	Lower incidence of severe mucositis

RT	Mascarin et al. (267)	Yes/yes/no	P	RT ± G-CSF	Less treatment interruptions only

RT	Schneider et al. (268)	Yes/yes/yes	P	RT ± G-CSF	Significant reduced incidence of severe mucositis

CT + RT	Bubley et al. (236)	Yes/yes/yes	P	Acyclovir vs. placebo	No impact upon incidence and severity of mucositis

*RT, radiotherapy; P/T, prevention or treatment; CT, chemotherapy; HD-CT, high-dose chemotherapy; BMT, bone marrow transplantation; TBI, total body irradiation; i.m., intramuscular; G-CSF, colony-stimulating factor; GM-CSF, granulocyte-macrophage colony-stimulating factor*.

*^a^Published with permission from Dr. Christoph C. Zielinski*.

### Clinical Trials for RIOM

Table 10 summarizes the clinical trials that were done until 2001 for prevention (P) and treatment (T) of RIOM (2). The current clinical trials for RIOM are summarized in Table 11 and were found when searching the clinical trials website of the National Institute of Health for RIOM. We have documented 40 RIOM treatment and prevention clinical trials.

**Table 11:** **Clinical trials for RIOM as listed on *http://www.ClinicalTrials.gov* when searched in November 2015**.

NCT number	Title	Conditions	Last updated
NCT02508389	A Study of GC4419 Protection against Radiation Induced Oral Mucositis in Patients with Head & Neck Cancer	Radiation-Induced Oral Mucositis	23 November 2015

NCT00698204	Cox-2 Inhibition in Radiation-Induced Oral Mucositis	Oral Mucositis	7 May 2014

NCT00814359	Magic Mouthwash Plus Sucralfate Versus Benzydamine Hydrochloride for the Treatment of Radiation-Induced Mucositis	Head and Neck Cancer|Mucositis	19 January 2011

NCT01400620	Safety and Efficacy of IZN-6N4 Oral Rinse for the Prevention of Oral Mucositis in Patients with Head and Neck Cancer	Oral Mucositis	9 November 2015

NCT00051441	Safety & Efficacy Study of Benzydamine Oral Rinse for the Treatment of Oral Mucositis (Mouth Sores) Resulting From Radiation Therapy for Cancer of the Oral Cavity, Oropharynx, or Nasopharynx	Stomatitis|Radiation Effects	17 May 2011

NCT02608879	Oral Care Protocol for the Management of Chemotherapy and Radiation Therapy-Induced Oral Mucositis	Oral Mucositis|Oral Cancer	17 November 2015

NCT01465308	The Effect of Honey on Xerostomia and Oral Mucositis	Head and Neck Cancer	7 October 2014

NCT01375088	Assessing the Preventing and Therapeutic Effect of Propolis in Radiotherapy Induced Mucositis of Head and Neck Cancers	Radiation-induced Mucositis of Oral Mucous Membranes	21 November 2012

NCT01066741	Prevention of Radiation-induced Severe Oral Mucositis in Oral Cavity, Oropharynx, Hypopharynx, and Cavum Cancer	Oropharynx Cancer|Hypopharynx Cancer	31 October 2012

NCT00006994	S9908: Glutamine in Treating Mucositis Caused by Radiation Therapy in Patients with Newly Diagnosed Cancer of the Mouth or Throat	Cancer-related Problem/Condition|Head and Neck Cancer|Pain	17 November 2015

NCT02430298	Topical/Oral Melatonin for Preventing Concurrent Radiochemotherapy Induced Oral Mucositis/Xerostomia Cancer Patients	Head and Neck Cancer	12 May 2015

NCT02397486	The Impact of Pentoxifylline and Vitamin E on Radiotherapy-induced Toxicity in Head & Neck Cancer Patients	Head and Neck Neoplasms	27 May 2015

NCT01941992	Role of SAMITAL^®^ in the Relief of Chemoradiation (CT-RT) Induced Oral Mucositis in Head and Neck Cancer Patients	Head-and-neck Squamous Cell Carcinoma|Oral Mucositis	24 March 2015

NCT01318889	Dexpanthenol Mouthwash to Treat Oral Mucositis	Oral Mucositis (Ulcerative) Due to Radiation	5 July 2011

NCT02016807	ZeroTolerance Mucositis: Managing Oral and Alimentary Mucositis with High Potency Sucralfate—ProThelial	Oral Mucositis|Nausea|Vomiting|Diarrhea	16 December 2013

NCT00293462	GM-CSF Mouthwash for Preventing and Treating Mucositis in Patients Who Are Undergoing Radiation Therapy for Head and Neck Cancer	Head and Neck Cancer|Mucositis|Radiation Toxicity	14 May 2013

NCT00728585	Palifermin in Preventing Oral Mucositis Caused by Chemotherapy and/or Radiation Therapy in Young Patients Undergoing Stem Cell Transplant	Breast Cancer|Graft vs. Host Disease|Kidney Cancer|Leukemia|Lymphoma|Mucositis|Multiple Myeloma|Plasma Cell Neoplasm|Myelodysplastic Syndromes|Neuroblastoma|Ovarian Cancer|Sarcoma|Testicular Germ Cell Tumor	30 May 2013

NCT02604329	Feasibility Study of a Protocol to Treat Pediatric Oral Mucositis by Low-Level Laser Therapy	Oral Mucositis	12 November 2015

NCT02075749	Comparing Triamcinolone Acetonide Mucoadhesive Films with Licorice Mucoadhesive Films	Mucositis	9 July 2014

NCT01385748	Efficacy and Safety Study of Clonidine Lauriad^®^ to Treat Oral Mucositis	Oral Mucositis	7 July 2015

NCT01707641	Effect of Lactobacillus Brevis CD2 in Prevention of Radio-chemotherapy Induced Oral Mucositis in Head and Neck Cancer	Mucositis	19 May 2014

NCT00613743	Effect of Topical Morphine (Mouthwash) on Oral Pain Due to Chemo- and/or Radiotherapy Induced Mucositis	Cancer|Mucositis	12 January 2010

NCT00431925	Can Cytokines Predict the Severity of Acute Mucositis and the Need for Gastrostomy Tubes (PEG)?	Oral Mucositis|Xerostomia|Weight Loss|Head and Neck Cancer	9 August 2007

NCT01806272	Recombinant Human Granulocyte Macrophage Colony-Stimulating Factor (rhGM-CSF) Treating Oral Mucositis	Nasopharyngeal Cancers	27 March 2013

NCT01876407	Effectiveness of Low Energy Laser Treatment in Oral Mucositis Induced by Chemotherapy and Radiotherapy in Head and Neck Cancer	Oral Mucositis	30 April 15

NCT00584597	A Trial of Homeopathic Medication TRAUMEEL S for the Treatment of Radiation-Induced Mucositis	Mucositis|Head and Neck Cancer	10 December 2010

NCT00615420	A Randomized Placebo-Controlled Trial of Manuka Honey for Oral Mucositis Due to Radiation Therapy for Cancer	Radiotherapy-Induced Mucositis|Head and Neck Cancer	22 May 2012

NCT01898091	Herbal Mouthrinse for Oral Mucositis Study	Oral Mucositis	2 1 Septmebr 2015

NCT01772706	Laser Mucite ORL: Effectiveness of Laser Therapy for Mucositis Induced by a Radio-chemotherapy in Head and Neck Cancer	Oral Squamous Cell Carcinoma|Squamous Cell Carcinoma of Oropharynx|Squamous Cell Carcinoma of Hypopharynx|Oral Mucositis	17 January 2013

NCT01837446	Morphine Mouthwash for Management of Oral Mucositis in Patients with Head and Neck Cancer	Stomatitis	22 April 2013

NCT02309437	Early Use of Opioid to Control Local Mucosa Pain Induced by Irradiation in Nasopharyngeal Carcinoma Patients	Nutrition Disorders|Quality of Life	3 December 2014

NCT01668849	Edible Plant Exosome Ability to Prevent Oral Mucositis Associated with Chemoradiation Treatment of Head and Neck Cancer	Head and Neck Cancer|Oral Mucositis	12 May 2015

NCT01975688	A Pharmacokinetic Study of Single Doses of Sativex in Treatment-Induced Mucositis	Head and Neck Squamous Cell Carcinoma	12 May 2015

NCT01252498	Evaluation of the Role of Prostaglandins in Radiation-induced Mucositis	Cancer of the Head and Neck|Radiotherapy	3 February 2014

NCT01840436	Efficacy of MUCIPLIQ on the Incidence of Radio-chemotherapy-Induced Mucositis in Patients Suffering From Oral Cancer	Oral Mucositis|Carcinoma *in Situ* of Upper Respiratory Tract	15 May 2014

NCT00699569	Hyperimmune Colostrum and Oral Mucositis	Head and Neck Cancer	22 July 2008

NCT02555501	Oral Mucositis and Laser Therapy Associated with Photodynamic Therapy	Oral Mucositis	18 September 2015

NCT02050503	Intranasal Transmucosal Fentanyl Pectin for Breakthrough Cancer Pain in Radiation-Induced Oropharyngeal Mucositis	Breakthrough Pain|Mucositis|Radiotherapy|Chemotherapy|Head and Neck Cancer	16 March 2015

NCT01883908	Acupuncture in Reducing the Severity of Chemoradiation-Induced Mucositis in Patients with Oropharyngeal Cancer	Mucositis|Oropharyngeal Cancer	3 September 2015

NCT01432873	Oral Selenium Therapy for the Prevention of Mucositis	Mucositis|Hematopoietic Stem Cell transplantation	31 May 2012

## Conclusion

Despite its high incidence, RIOM is a self-limited radiotherapy-induced normal tissue injury. It is a dose-limiting toxicity in most cases of head and neck cancer patients. However, in moderately to severely sick patients, it could be a lethal injury. Many preclinical and clinical studies have been conducted for the prevention and treatment of RIOM. Currently, there are numerous prevention and treatment strategies for RIOM. However, there is no single agent or management regimen that has been agreed upon between caregivers that significantly improves RIOM to a clinically relevant and satisfactory standard. Nevertheless, the current guidelines recommend good oral care, IMRT, radiation shields, palifermin, amifostine, and cryotherapy for RIOM prevention. RIOM treatment focuses on palliative measures and symptoms relief; e.g., pain management, nutritional support, good oral hygiene, and reduced oral microbial load. Interestingly, mesenchymal stromal cells therapy for RIOM shows promise for potential therapeutic and clinically relevant benefits. However, more studies are still needed to confirm such therapeutic potential.

## Author Contributions

OM: conception and design, collection and/or assembly of data, review writing, and final approval of the review. NE: conception, design, and final approval of the review. TM: conception and design, financial support, and final approval of the review.

## Conflict of Interest Statement

The authors declare that the research was conducted in the absence of any commercial or financial relationships that could be construed as a potential conflict of interest.
